# Bactericidal Effects and Quality Impact of Peroxyacetic Acid and Sodium Hypochlorite on Chicken Carcasses

**DOI:** 10.3390/foods13081204

**Published:** 2024-04-15

**Authors:** Bo-Zheng Zhang, Jin-Man Kim, Jung-Min Park

**Affiliations:** Department of Food Marketing and Safety, Konkuk University, Seoul 05029, Republic of Korea; slurpee24@163.com (B.-Z.Z.);

**Keywords:** peroxyacetic acid, sodium hypochlorite, decontaminant, poultry production, chicken carcasses

## Abstract

There is an urgent need to develop efficient and environmentally friendly decontaminants for poultry products. In this study, we aimed to evaluate the practical application of peroxyacetic acid (PAA) as a replacement for sodium hypochlorite (SH) to sterilize fresh chicken carcasses, using microbial, color, and electronic-nose analyses. We evaluated the decontamination effects of different concentrations of PAA and SH on chicken carcasses. The bactericidal effects of PAA at pH 3, 7, and 9, and SH at pH 10, at concentrations ranging from 100 to 500 ppm on coliform bacteria, total bacteria, and *Salmonella* spp. were evaluated. PAA induced a similar bactericidal effect at lower concentrations than SH. Therefore, at the same concentration and treatment time, PAA showed better bactericidal effects than SH. Although treatment with PAA (pH 3) and SH (pH 10) resulted in considerable discoloration, the degree of discoloration decreased when the pH of PAA was increased to 7 and 9. Therefore, by increasing the pH of PAA, the discoloration effect on chicken carcasses can be reduced without altering the microbial-reduction effect. Electronic-nose analysis showed that the flavor of the chicken was almost unaffected by volatile components at a treatment time < 30 min. Therefore, this study experimentally identified the optimal PAA concentration for the decontamination of chicken carcasses. The study findings provide a theoretical basis for the replacement of traditional bactericides, such as SH, with PAA for the production of poultry products.

## 1. Introduction

Food-consumption patterns have evolved in recent years, with the per capita chicken consumption in South Korea showing an upward trend [1]. Sales of pre-packaged chicken in large discount markets have grown rapidly; however, issues, such as a short shelf life, improper storage, and easy deterioration, remain. Chickens are exposed to feces during the slaughter process, and this is a major source of microbial contamination. Moreover, the internal organs and intestinal contents of the chicken carcass can also contaminate the chicken during processing. Thus, without prompt decontamination, they rapidly rot and deteriorate and may cause foodborne outbreaks.

Slaughtered chicken carcasses carry numerous pathogens. Mishandling of the carcasses may result in deterioration due to microorganisms, foodborne outbreaks after consumption, and serious economic losses. This is an important public health issue and a major concern for the food industry [2]. Therefore, decontamination should be performed immediately after slaughter to minimize the risk of foodborne outbreaks and prolong the shelf life of chicken [3].

The use of decontaminants is a means of ensuring the safety of poultry products. In Korea, the primary decontaminant currently used is sodium hypochlorite (SH) [4]. The primary concern with the use of chlorine-based decontaminants is the reaction of chlorine with organic matter, which often results in decontamination by-products (DBPs) that create health hazards. DBPs mainly include trihalomethanes (THMs), haloacetic acids (HAAs), carbonaceous DBPs (C-DBPs), nitrogenous DBPs (N-DBPs), nitrosamines (NISAMs), and aldehydes. DBP formation consumes the decontaminant, gradually reducing its bactericidal effect, and leads to an increased possibility for the microbial contamination of food [5]. Therefore, an efficient and environmentally friendly decontaminant for poultry products is urgently required.

Peroxyacetic acid (PAA) is such a decontaminant. Its decomposition does not produce by-products that affect the human body or environment. Generally, PAAs are used to sterilize food and beverages, cooling-tower water, wastewater, and stormwater [6]. In 2018, PAA was repurposed as a food sanitizer in Korea [7]. The maximum PAA concentration used in South Korea for handling poultry is 2000 ppm [8]. PAA is more efficient and stable than traditional biocide chlorine over a wide pH range [9], and can decompose directly in air without an additional removal process after its use. Even if by-products are generated, they are non-toxic [10].

Although PAA is used to decontaminate chickens in some countries, further research is required on the variation in decontamination efficiency with respect to procedures, concentrations, treatment times, and pathogen types. PAA does not produce residual toxicity, but it does have a sour taste that may affect the flavor of chicken, and this factor should be evaluated by analyzing the volatile components of treated chicken using headspace electronic-nose technology [11,12].

Traditional sensory evaluation can objectively assess the taste, smell, and visual perception of human beings on related foods and directly solve production problems for food-industry enterprises in a timely manner [13,14]. Although disadvantages, such as poor reproducibility and large individual differences, remain, the electronic nose of simulated bionic detection equipment can compensate for the disadvantages of sensory detection [15]. Electronic-nose analysis primarily uses sensor and pattern-recognition technology to analyze the “information profile” of complex systems in a more intuitive way. With further research and development, electronic-nose detection and analysis technology will play key roles in the food and pharmaceutical industries. This technology assists in discrimination and identification analysis, freshness discrimination, and maturity determination [16,17].

In addition, some studies have shown that PAA changes the color of meat when used [18]. Therefore, in this study, the color of chicken before and after PAA treatment was tested and compared through a color difference experiment, and the occurrence of discoloration was controlled by adjusting the pH value as much as possible while ensuring decontamination.

Various decontaminant products currently used in slaughterhouses have limitations, thus replacing them with more environmentally friendly and effective decontaminants is necessary. Because South Korea designated PAA as a food decontaminant only in 2018, domestic research on the effects of PAA on chickens remains limited. The upper limit of PAA that can be used has been specified, but the specific working concentration has not yet been defined, which is detrimental for cost-effectiveness and the environment. Therefore, this study aimed to evaluate the practical application of PAA using microbial, color, and electronic-nose analyses and to determine its applicability as a replacement for SH. Our findings provide useful information for the slaughtering and processing of poultry products.

## 2. Materials and Methods

### 2.1. Chicken-Carcass Samples

The chicken-carcass samples were obtained from a provincial factory (Jincheon-gun, Chungcheongbuk-do, Republic of Korea) and arrived at our laboratory 24 h after cold-chain (4 °C) delivery. They were stored at 4 °C until further analysis. More than 150 chicken carcasses weighing 2.0 ± 0.2 kg, with roughly the same shape and no heads or feet, were screened in the slaughterhouse for this experiment. Among them, 70 were used in the microbial experiment, 70 were used in the chromatic aberration experiment, and 15 were used in the electronic nose experiment. To control the variables, all chickens, the carcasses of which were used in this experiment, were slaughtered using the same method and were from the same production line. All chicken carcasses were from the same lot on the same day. To compare the effects of the various decontamination treatments after slaughter, a microbial control was not performed. To ensure the accuracy and reliability of the data, all experimental procedures and data analyses were performed in the BSL-2 laboratory by professional personnel.

### 2.2. Preparation of Decontaminants and Washing Conditions

PAA and SH were used as the decontaminants. PAA (16%) was obtained from Daesung Co. (Seoul, Republic of Korea) and SH (5.5%) was obtained from Yuhanclorox Co. (Seoul, Republic of Korea). PAA and SH solutions were diluted to concentrations of 100, 200, 300, 400, and 500 ppm. The pH of directly diluted PAA was approximately 3, whereas that of directly diluted SH was approximately 10. To compare the effects of different pH conditions, a sodium hydroxide solution was used to adjust the pH of PAA to 7 and 9. All solutions were prepared using sterile distilled water at room temperature (25 °C) on the day of the experiment.

### 2.3. Microbial Analysis of Chicken Carcass Samples

For microbiological analysis, chicken carcasses were immersed in decontaminant buckets (~6 L) and soaked for 15, 30, and 45 min. PAA (pH 3, 7, and 9) and SH (pH 10) decontaminants at concentrations of 100, 200, 300, 400, and 500 ppm were used. Experiments were performed in triplicate at each pH and at each concentration, using a total of 60 chicken carcasses. A control group of three chicken carcasses was briefly soaked in sterile deionized water. From the treated chicken carcasses, using sterile scissors, 5 g chicken breast was collected from the same sampling area and depth of each sample. The samples contained chicken skin. The samples were mixed with 45 g of peptone water in a homogenizing bag, and processed using a homogenizer (Stomacher 400 Circulator, Seward Inc., West Sussex, UK) at 300 rpm for 2 min. The homogenate was then transferred to a 1.5 mL centrifuge tube and stored until microbial analysis.

To quantify the number of microorganisms, the samples were diluted to 10^2^–10^4^, and 0.1 mL was inoculated onto the surface of a flat medium plate, spread evenly, and incubated. Homogenized diluted samples were inoculated on eosin methylene blue agar (EMB; Difco, BD, Detroit, MI, USA), xylose lysine deoxycholate agar (XLD; Difco, BD, Detroit, MI, USA), and plate count agar (PCA; Difco, BD, Detroit, MI, USA) and used to count coliforms, *Salmonella* spp., and total bacterial counts, respectively. EMB Agar, XLD Agar, and PCA were cultured at 37 °C for 24 h, and then colonies were counted.

### 2.4. Color Measurement

Before decontamination, the chromaticity of the wing tip, neck, and breast of the entire chicken was measured thrice using a colorimeter, and the results were used as the control. The colors of the same parts were measured after soaking and decontamination. We used a colorimeter (LICO690, Hash, Loveland, CO, USA) to measure the L* (luminance axis), a* (red–green axis), and b* (yellow–blue axis) of the sample. The color difference was then calculated using the following formula: ∆E* = ((∆L*)^2^ + (∆a*)^2^ + (∆b*)^2^)^0.5^. The influence of each decontaminant at each concentration and pH on the color change of the chicken was determined [19].

### 2.5. Electronic-Nose Analysis

PAA residues in the chicken-carcass samples were analyzed using a HERACLES II Electronic Nose (Alpha MOS, Toulouse, France). The samples were treated with 100, 300, and 500 ppm PAA (pH 3) for 15, 30, and 45 min; the control group was untreated chicken carcasses. For statistical analysis, the experiment was repeated three times for each concentration condition. Using the same sampling method as for microbiological experiments, each 5 g sample was placed in a glass jar, sealed, and refrigerated at 4 °C. The samples were processed under the following conditions: heating temperature, 60 °C; flow rate, 250 mL/min; injection volume, 2.5 mL; acquisition time, 120 s; and trapping temperature, 40 °C/240 °C. The sample volatile components (Rair) were analyzed for the rate of change in resistance (Rgas) using AlphaSoft software (version 11; Alpha MOS, Toulouse, France) for the principal component analysis (PCA) of each sensor. These measured odor components were represented in a PCA plot, and primary (PC1) and secondary (PC2) component values were obtained to differentiate the fragrance patterns. PCA was used to simplify datasets. This was achieved by retaining the lower-order principal components and ignoring higher-order principal components. Thus, lower-order components tend to retain the most important aspects of the data. However, this is not a requirement, and depends on the application [20,21].

### 2.6. Statistical Analysis

Each experiment was performed in triplicate. Data were analyzed using the SPSS software (version 25.0; SPSS, Inc., Chicago, IL, USA), and a one-way analysis of variance (ANOVA) was performed to determine significance. Duncan’s multiple range test was used to identify significant differences between treatments. A *p*-value < 0.05 was considered to be statistically significant. The electronic-nose experiment was analyzed by principal component analysis (PCA) to determine significance.

## 3. Results and Discussion

### 3.1. Microbial Analysis of Chicken Carcasses

#### 3.1.1. Reduction in Coliform Bacteria

The decontamination efficiencies of PAA and SH against bacterial coliforms on the chicken carcasses are shown in Table 1. The reduction in the coliform load of chicken treated for 15 min with 100 ppm PAA (pH 3, 7, and 9) ranged from 1.12 to 1.35 log_10_CFU/mL, while that in chicken treated with SH (pH 10) was of 0.24 log_10_CFU/mL. In the 200, 300, 400, and 500 ppm PAA and SH treatments, the coliform load reductions were 1.54–1.75, 1.72–1.89, 1.95–2.07, and 2.18–2.34 and 0.31, 0.48, 0.79, and 0.97 log_10_CFU/mL, respectively. At higher concentrations, the difference in the bactericidal effect between PAA and SH was similar to the results at 100 ppm. After treating the chicken carcasses for 30 min with 100, 200, 300, 400, and 500 ppm PAA at each pH (3, 7, and 9), the reduction in the coliform load was of 1.57–1.70, 1.91–2.09, 2.11–2.20, 2.24–2.46, and 2.46–2.63 log_10_CFU/mL, respectively. Coliform reduction in chicken carcasses treated with SH (100, 200, 300, 400, and 500 ppm; pH 10) for 30 min ranged from 0.37 to 1.23 log_10_CFU/mL.

The chicken carcasses treated for 45 min with 100, 200, 300, 400, and 500 ppm PAA at each pH (3, 7, and 9) had a reduction in the coliform load of 1.77–2.03, 2.21–2.47, 2.52–2.69, 2.62–2.90, and 2.88–3.15 log_10_CFU/mL, respectively. The reduction in coliform load in the chicken carcasses treated with SH (100, 200, 300, 400, and 500 ppm; pH 10) for 45 min ranged from 0.49 to 1.51 log_10_CFU/mL.

Thus, the bactericidal effect of PAA increased with increasing concentrations. At a constant PAA concentration, an increase in the treatment time resulted in a significant difference in the bactericidal effect (*p* < 0.05). This is similar to the experimental results reported by Thomas et al. and Ramirez-Hernandez et al. [22,23]. However, no significant differences were observed at different PAA pH values, indicating that PAA maintains a stable bactericidal effect over a wide pH range. This result is similar to that reported by Chaplot et al. [24].

#### 3.1.2. Reduction in Total Viable Counts

The bactericidal effects of PAA (pH 3, 7 and 9) and SH (pH 10) on general bacteria in the chicken carcasses are shown in Table 2. When entire chicken was treated for 15 min with 100 ppm PAA and SH, the general bacterial-load reduction was 1.02–1.07 log_10_CFU/mL and 0.29 log_10_CFU/mL, respectively. In the 200, 300, 400, and 500 ppm PAA and SH treatments, the coliform load reduction was 1.39–1.55, 1.60–1.73, 1.81–1.92, and 1.98–2.09 and 0.51, 0.58, 0.65, and 0.82 log_10_CFU/mL, respectively. Therefore, the bactericidal effect of 500 ppm SH was similar to that of 100 ppm PAA. Based on the varying pH, the general bacterial-load reductions in all chickens after treatment with PAA (pH 3, 7, and 9) for 30 min at 100, 200, 300, 400, and 500 ppm were 1.30–1.37, 1.64–1.80, 2.05–2.15, 2.21–2.32, and 2.30–2.46 log_10_CFU/mL, respectively, while the bacterial-load reductions of SH (100, 200, 300, 400, and 500 ppm; pH 10) were 0.40–1.38 log_10_CFU/mL. After treatment with PAA (pH 3, 7, and 9) for 45 min at 100, 200, 300, 400, and 500 ppm, the load reductions of general bacteria in chicken carcasses were 1.44–1.76, 2.08–2.30, 2.24–2.38, and 2.44–2.56 and 2.73–2.91 log_10_CFU/mL, respectively, while the general bacterial-load reductions in chicken carcasses after treatment with SH (100, 200, 300, 400, and 500 ppm; pH 10) were 0.45–1.43 log_10_CFU/mL. These findings suggest that the bactericidal effect of PAA on general bacteria is marginally weaker than that on coliforms. However, a trend was observed between the general bacteria and coliforms. This is similar to the experimental results of Rilana et al. [25]. Thus, the bactericidal effect of PAA on general bacteria increased significantly with increasing PAA concentration (*p* < 0.05). However, no significant differences were observed at different pH values, indicating that PAA maintained a stable bactericidal effect against bacteria over a wide pH range.

One of the main problems associated with the use of chlorine-based decontaminants is the reactivity of chlorine with organic compounds, as it can create DBPs with potential health hazards. A study by Lee [5] showed that the decontamination process using SH produces more byproducts (DBPs) than that using PAA, and that the reaction that leads to the formation of DBPs depletes the decontaminant and reduces its efficacy in inactivating pathogens, increasing the chance of microbial contamination in food. Compared with SH, PAA produces fewer DBPs during continuous decontamination and can consistently provide a stable and efficient decontamination environment. 

#### 3.1.3. Reduction in *Salmonella* spp.

*Salmonella* spp. were not detected on the chicken carcasses after 15–45 min of treatment with 100–500 ppm PAA, and they were also absent in the control group. According to the regulations of the Ministry of Food and Drug Safety, *Salmonella* spp. should be absent from poultry. The findings of the present study are consistent with these guidelines.

### 3.2. Analysis of Color Differences in Chicken Carcasses

#### 3.2.1. Chicken Wings

The results of treating chicken wings with the same concentrations of PAA and SH are presented in Table 3. At pH 3, the color differences of chicken-wing tips, treated with different PAA concentrations (100, 200, 300, 400, and 500 ppm) and treatment times (15, 30, and 45 min), ranged from 4.25 to 14.45. At similar PAA concentrations and treatment times, the color differences at pH 7 and 9 ranged from 2.14 to 11.07 and 2.74 to 9.73, respectively. The experimental results of Bauermeister et al. [26] and Olson et al. [27] also showed that chickens had different degrees of discoloration after exposure to PAA at different concentrations. After treatment with SH (pH 10), the color difference ranged from 6.49 to 10.71. Similarly, the experimental results of Lim et al. [28] showed that treatment of meat with sodium hypochlorite changed the meat color. Thus, in most cases, SH at pH 10 has a similar effect to PAA at pH 3, and the change in chicken-carcass color is much greater than that of PAA at pH 7 and 9. However, the discoloration effect on chicken wings at pH 7 was significantly reduced at the same concentration. When the pH of PAA was 9, the degree of discoloration of the chicken wings was lower than that at pH 7. Thus, when PAA is used to sterilize chickens, the color change of chicken wings is minimally affected under alkaline conditions. The discoloration of chicken wings continued to increase with increasing treatment time (*p* < 0.05).

#### 3.2.2. Chicken Breast

The results of the chicken-breast treatment are presented in Table 3. At the same concentration, treatment with PAA at pH 3 and SH at pH 10 showed relatively more discoloration, with color difference values of 2.26–13.58 and 2.83–13.20, respectively. For each PAA treatment concentration, the degree of discoloration was the lowest at pH 9. Therefore, the results are similar to those obtained for the chicken wings. The appearance of chicken breasts was minimally affected after PAA treatment under alkaline conditions. The discoloration of chicken breasts continued to increase with increasing treatment time (*p* < 0.05).

#### 3.2.3. Chicken Necks

The results of the chicken-neck treatment are presented in Table 3. After treatment with PAA at pH 9, the degree of discoloration was the lowest, with color difference values ranging from 2.23 to 8.80. Similar to chicken wings and breasts, sanitizing chickens with alkaline PAA had a minimal impact on the appearance of the chicken necks. The ionic action of the fungicide on the surface of the chicken caused the protein to solidify and the chicken to change color. By adjusting the pH value and reducing the ionic effect, this effect can be significantly suppressed. However, the degree of discoloration of the chicken neck continued to increase (*p* < 0.05) with increasing in treatment time, and due to the lack of skin, the degree of discoloration of the chicken neck was much higher than that of the wings and breasts in most cases. The experimental results of Yang et al. [29] showed that chicken necks were more discolored during handling than the other parts.

### 3.3. Electronic-Nose Analysis

**Figure 1:**
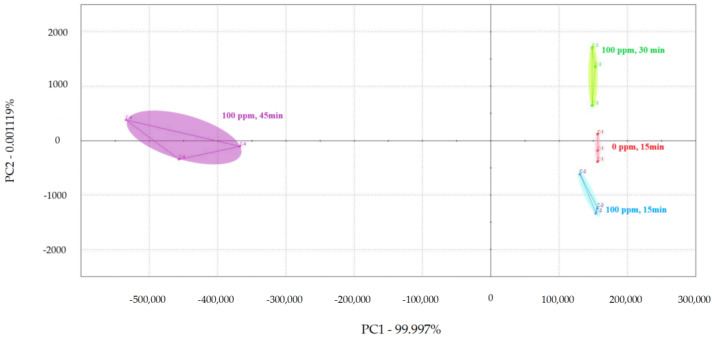
Principal component analysis plot of chicken carcasses on treatment with 100 ppm peroxyacetic acid.

**Figure 2:**
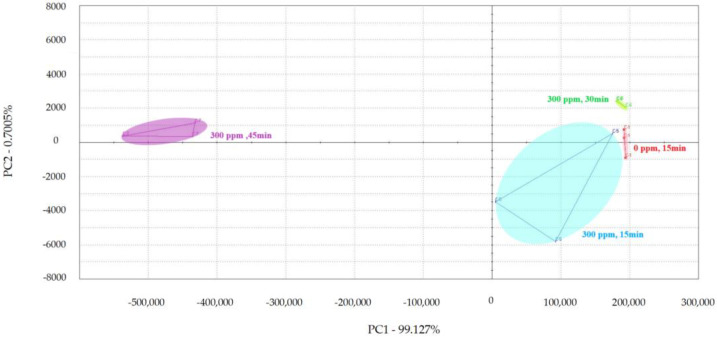
Principal component analysis plot of chicken carcasses on treatment with 300 ppm peroxyacetic acid.

**Figure 3:**
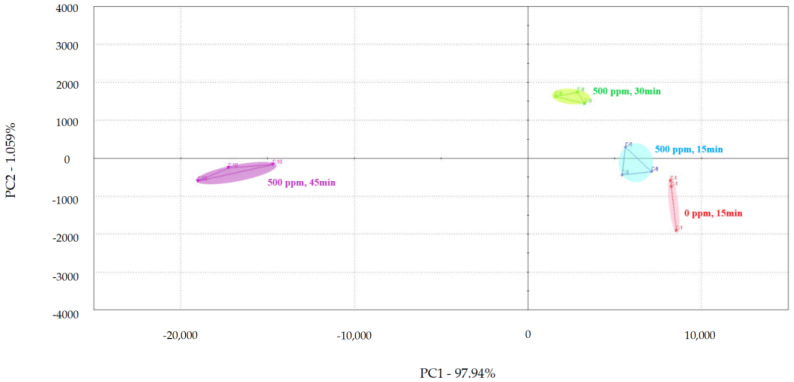
Principal component analysis plot of chicken carcasses on treatment with 500 ppm peroxyacetic acid.

PCA was used to examine the impact of different treatment times on the volatile flavor of chicken (Figure 1, Figure 2 and Figure 3) [30,31,32]. After the sensor-response data were processed using the fractionation method, they was classified using PCA. In this study, the score plot of PC1 versus PC2 for chicken carcasses treated with 100, 300, and 500 ppm PAA according to treatment time was analyzed using electronic-nose technology, as shown in Figure 1, Figure 2 and Figure 3. However, in the score plot, PAA-treated chicken carcasses at each treatment time (15, 30, and 45 min) could be clearly separated by the electronic nose. The contribution rates of PC2 at 100, 300, and 500 ppm of PAA were 0.0011%, 0.7005%, and 1.0590%, respectively, which were lower than the respective PC1 values of 99.997%, 99.127%, and 97.940%. A study by Balaban [33] showed that the total contribution rate of the principal components was >95%, indicating that this method was suitable. In this experiment, the contribution rate of the principal components met the above requirements; thus, the PCA analysis of electronic-nose results better distinguished the differences in chicken flavor under different treatment conditions. The high contribution rate of PC1 compared to PC2 was valuable, because the difference between the corresponding *x*-axis treatment spheres with respect to PC1 is a measure of flavor difference [34,35]. Differences in PC1 (*x*-axis) among the three treatment conditions varied with the treatment time at the same concentration, with no significant differences observed after 15 and 30 min of treatment. PAA eventually decomposed over time after treatment without leaving toxic residues in the material [36]. However, at 45 min, a considerable difference was observed compared to the control group. Although acetic acid evaporated rapidly after treatment, trace amounts remained. Increasing the treatment time increases the residual PAA content in the sample. Thus, the treatment time is recommended to be within 30 min to minimize residual PAA and ensure that the chicken flavor remains unchanged. Rezende et al. [37] confirmed that treating chicken with PAA for extended periods can lead to residue in the chicken.

According to the electronic-nose-analysis results, after PAA treatment, the flavor effect on chickens changed with increasing treatment time, particularly after 30 min. Although PAA decomposes in air and does not affect the edibility of the treated chicken, it remained in the chicken for a short time after processing. Therefore, the appropriate decontamination time using an electronic-nose experiment must be determined to minimize the influence of PAA on chicken flavor under effective decontamination conditions. Punchihewage-Don et al. [38] showed that high concentrations of PAA can affect chicken flavor. This study shows that electronic-nose technology can classify PAA-treated chicken carcasses according to the contact time, even though the samples had similar aromas.

Decontaminants that effectively reduce microbial contamination of poultry products are critical for protecting poultry supply chains. For general bacteria and coliforms carried by chicken carcasses, the use of PAA can effectively reduce their numbers by 2–3 log_10_CFU/mL, whereas the same concentration of SH can only reduce it by 1–2 log_10_CFU/mL. Thames et al. [39] and Andreoletti et al. [40] showed similar experimental results. Therefore, during the production of chicken carcasses, PAA, with its excellent sterilization ability, can effectively replace SH and reduce the risk of foodborne outbreaks. In addition, PAA can be used over a wide pH range, because changing the pH barely affects its bactericidal properties.

We also analyzed the color differences in chicken carcasses after treatment. Upon soaking the chicken carcasses in PAA, the color of the wing tips, breasts, and necks changed significantly with an increase in treatment time from 15 to 45 min. However, an increase in the pH of PAA from 3 to 9 showed that the effect of alkaline conditions on the color of chicken carcasses was significantly lower than that under acidic conditions. In most cases, the effect of SH at pH 10 on chicken color was similar to that of PAA at pH 3. However, at the same concentrations, the bactericidal effect of PAA was significantly greater than that of SH. In addition, the electronic-nose experimental results showed that the flavor of the chicken changed as the treatment time increased from 15 to 45 min. Under the same experimental conditions, we believe that the change in the flavor of chicken meat was caused by an increase in residual PAA with increasing treatment time. Interestingly, when PAA was used to sterilize chicken carcasses in this experiment, the soaking time was <30 min, and there was almost no effect on the flavor of the chicken. However, when the soaking time reached 45 min, the flavor of the chicken was significantly affected.

## 4. Conclusions

In conclusion, PAA is more efficient and safer than the traditional decontaminant, SH. In this study, PAA quickly and effectively reduced the number of general and coliform bacteria and significantly inhibited the color change of chicken meat during sterilization at high pH values. Retaining the original color of chicken may make customers more eager to buy it. The experimental electronic-nose results showed that the treatment time should be ≤ 30 min when using PAA to treat chicken to prevent the flavor-change effect. This study provides a valuable reference for chicken production and processing, as our findings suggest that PAA, as a novel food surface decontaminant, has the potential to replace traditional fungicides, such as SH. However, further research is required to determine the optimal conditions for its application at a production scale.

## Figures and Tables

**Table 1:** The effect of treatment time with peroxyacetic acid (PAA) and sodium hypochlorite (SH) on coliform load.

ppm	Decontaminant	pH	Coliform Load (log_10_CFU/mL)			
			Treatment Time (min)			
			0	15	30	45
100	Peroxyacetic Acid	3	6.02 ± 0.07 ^Aa^	4.67 ± 0.06 ^Bc^	4.36 ± 0.11 ^Cb^	3.99 ± 0.11 ^Dc^
		7	6.02 ± 0.07 ^Aa^	4.76 ± 0.09 ^Bbc^	4.32 ± 0.11 ^Cb^	4.13 ± 0.14 ^Cbc^
		9	6.02 ± 0.07 ^Aa^	4.90 ± 0.05 ^Bb^	4.45 ± 0.10 ^Cb^	4.25 ± 0.11 ^Db^
	Sodium Hypochlorite	10	6.02 ± 0.07 ^Aa^	5.78 ± 0.12 ^Ba^	5.65 ± 0.16 ^BCa^	5.53 ± 0.09 ^Ca^
200	Peroxyacetic Acid	3	6.02 ± 0.07 ^Aa^	4.27 ± 0.09 ^Bc^	3.93 ± 0.10 ^Cb^	3.55 ± 0.15 ^Dc^
		7	6.02 ± 0.07 ^Aa^	4.40 ± 0.13 ^Bbc^	4.06 ± 0.09 ^Cb^	3.81 ± 0.11 ^Db^
		9	6.02 ± 0.07 ^Aa^	4.48 ± 0.08 ^Bb^	4.11 ± 0.17 ^Cb^	3.73 ± 0.09 ^Dbc^
	Sodium Hypochlorite	10	6.02 ± 0.07 ^Aa^	5.71 ± 0.07 ^Ba^	5.38 ± 0.08 ^Ca^	5.19 ± 0.11 ^Da^
300	Peroxyacetic Acid	3	6.02 ± 0.07 ^Aa^	4.19 ± 0.10 ^Bb^	3.82 ± 0.09 ^Cb^	3.33 ± 0.10 ^Db^
		7	6.02 ± 0.07 ^Aa^	4.13 ± 0.11 ^Bb^	3.85 ± 0.03 ^Cb^	3.50 ± 0.12 ^Db^
		9	6.02 ± 0.07 ^Aa^	4.30 ± 0.10 ^Bb^	3.91 ± 0.04 ^Cb^	3.48 ± 0.13 ^Db^
	Sodium Hypochlorite	10	6.02 ± 0.07 ^Aa^	5.54 ± 0.14 ^Ba^	5.19 ± 0.10 ^Ca^	5.02 ± 0.08 ^Ca^
400	Peroxyacetic Acid	3	6.02 ± 0.07 ^Aa^	4.00 ± 0.08 ^Bb^	3.56 ± 0.05 ^Cc^	3.12 ± 0.15 ^Dc^
		7	6.02 ± 0.07 ^Aa^	3.95 ± 0.08 ^Bb^	3.71 ± 0.07 ^Cbc^	3.30 ± 0.12 ^Dbc^
		9	6.02 ± 0.07 ^Aa^	4.07 ± 0.12 ^Bb^	3.78 ± 0.11 ^Cb^	3.40 ± 0.07 ^Db^
	Sodium Hypochlorite	10	6.02 ± 0.07 ^Aa^	5.23 ± 0.13 ^Ba^	5.03 ± 0.11 ^Ba^	4.73 ± 0.13 ^Ca^
500	Peroxyacetic Acid	3	6.02 ± 0.07 ^Aa^	3.71 ± 0.05 ^Bb^	3.52 ± 0.08 ^Cbc^	2.87 ± 0.08 ^Dc^
		7	6.02 ± 0.07 ^Aa^	3.68 ± 0.09 ^Bb^	3.39 ± 0.11 ^Cc^	2.99 ± 0.11 ^Dbc^
		9	6.02 ± 0.07 ^Aa^	3.84 ± 0.09 ^Bb^	3.56 ± 0.07 ^Cb^	3.14 ± 0.10 ^Db^
	Sodium Hypochlorite	10	6.02 ± 0.07 ^Aa^	5.05 ± 0.19 ^Ba^	4.79 ± 0.07 ^Ca^	4.51 ± 0.10 ^Da^

Values are presented as the mean ± standard deviation (log_10_CFU/mL) (*n* = 3). The detection limit was 1.00 log_10_CFU/mL. Different uppercase letters in the same row (A–D) indicate significant differences (*p* < 0.05) according to Duncan’s multiple range test. Different lowercase letters in the same column (a–c) indicate significant differences (*p* < 0.05) according to Duncan’s multiple range test.

**Table 2:** The effect of treatment time with PAA and SH on the reduction in total bacterial load.

ppm	Decontaminant	pH	Total Bacterial Load (log_10_CFU/mL)			
			Treatment Time (min)			
			0	15	30	45
100	Peroxyacetic Acid	3	6.93 ± 0.06 ^Aa^	5.91 ± 0.11 ^Bb^	5.56 ± 0.04 ^Cb^	5.17 ± 0.07 ^Dc^
		7	6.93 ± 0.06 ^Aa^	5.86 ± 0.06 ^Bb^	5.61 ± 0.09 ^Cb^	5.33 ± 0.11 ^Dbc^
		9	6.93 ± 0.06 ^Aa^	5.90 ± 0.12 ^Bb^	5.63 ± 0.12 ^Cb^	5.49 ± 0.10 ^Cb^
	Sodium Hypochlorite	10	6.93 ± 0.06 ^Aa^	6.64 ± 0.13 ^Ba^	6.53 ± 0.14 ^Ba^	6.48 ± 0.08 ^Ba^
200	Peroxyacetic Acid	3	6.93 ± 0.06 ^Aa^	5.54 ± 0.11 ^Bb^	5.19 ± 0.13 ^Cb^	4.63 ± 0.16 ^Db^
		7	6.93 ± 0.06 ^Aa^	5.50 ± 0.13 ^Bb^	5.29 ± 0.10 ^Cb^	4.79 ± 0.07 ^Db^
		9	6.93 ± 0.06 ^Aa^	5.38 ± 0.11 ^Bb^	5.13 ± 0.10 ^Cb^	4.85 ± 0.12 ^Db^
	Sodium Hypochlorite	10	6.93 ± 0.06 ^Aa^	6.42 ± 0.09 ^Ba^	6.20 ± 0.08 ^Ca^	6.15 ± 0.13 ^Ca^
300	Peroxyacetic Acid	3	6.93 ± 0.06 ^Aa^	5.33 ± 0.12 ^Bb^	4.84 ± 0.10 ^Cb^	4.55 ± 0.06 ^Db^
		7	6.93 ± 0.06 ^Aa^	5.20 ± 0.14 ^Bb^	4.88 ± 0.08 ^Cb^	4.60 ± 0.14 ^Db^
		9	6.93 ± 0.06 ^Aa^	5.24 ± 0.08 ^Bb^	4.78 ± 0.07 ^Cb^	4.69 ± 0.08 ^Cb^
	Sodium Hypochlorite	10	6.93 ± 0.06 ^Aa^	6.35 ± 0.07 ^Ba^	5.82 ± 0.10 ^Ca^	5.70 ± 0.15 ^Ca^
400	Peroxyacetic Acid	3	6.93 ± 0.06 ^Aa^	5.11 ± 0.12 ^Bb^	4.69 ± 0.13 ^Cb^	4.37 ± 0.13 ^Db^
		7	6.93 ± 0.06 ^Aa^	5.01 ± 0.17 ^Bb^	4.61 ± 0.12 ^Cb^	4.41 ± 0.10 ^Cb^
		9	6.93 ± 0.06 ^Aa^	5.12 ± 0.19 ^Bb^	4.72 ± 0.07 ^Cb^	4.49 ± 0.08 ^Db^
	Sodium Hypochlorite	10	6.93 ± 0.06 ^Aa^	6.28 ± 0.11 ^Ba^	5.71 ± 0.16 ^Ca^	5.62 ± 0.16 ^Ca^
500	Peroxyacetic Acid	3	6.93 ± 0.06 ^Aa^	4.92 ± 0.12 ^Bb^	4.57 ± 0.15 ^Cb^	4.02 ± 0.16 ^Db^
		7	6.93 ± 0.06 ^Aa^	4.84 ± 0.09 ^Bb^	4.47 ± 0.16 ^Cb^	4.20 ± 0.11 ^Db^
		9	6.93 ± 0.06 ^Aa^	4.95 ± 0.10 ^Bb^	4.63 ± 0.12 ^Cb^	4.12 ± 0.10 ^Db^
	Sodium Hypochlorite	10	6.93 ± 0.06 ^Aa^	6.11 ± 0.09 ^Ba^	5.55 ± 0.13 ^Ca^	5.50 ± 0.16 ^Ca^

Values are presented as the mean ± standard deviation (log_10_CFU/mL) (*n* = 3). The detection limit was 1.00 log_10_CFU/mL. Different uppercase letters in the same row (A–D) indicate significant differences (*p* < 0.05) according to Duncan’s multiple range test. Different lowercase letters in the same column (a–c) indicate significant differences (*p* < 0.05) according to Duncan’s multiple range test.

**Table 3:** Effect of PAA and SH at different concentrations on color differences of chicken carcasses.

Concentration			∆E*			
			PAA—pH 3	PAA—pH 7	PAA—pH 9	SH—pH 10
Chicken wings	100 ppm	15 min	4.26 ± 0.27 ^cA^	2.25 ± 0.10 ^cB^	2.75 ± 0.64 ^cB^	2.93 ± 0.14 ^bB^
		30 min	6.61 ± 0.40 ^bA^	5.48 ± 0.21 ^bB^	4.59 ± 0.23 ^bC^	3.83 ± 0.73 ^bC^
		45 min	7.94 ± 0.18 ^aA^	7.29 ± 0.27 ^aB^	6.55 ± 0.27 ^aC^	6.52 ± 0.41 ^aC^
	200 ppm	15 min	5.20 ± 0.59 ^cA^	2.75 ± 0.42 ^cB^	3.03 ± 0.54 ^cB^	3.34 ± 0.60 ^cB^
		30 min	7.46 ± 0.14 ^bA^	7.07 ± 0.29 ^bA^	4.80 ± 0.19 ^bB^	5.30 ± 0.69 ^bB^
		45 min	9.03 ± 0.25 ^aA^	8.54 ± 0.12 ^aB^	7.48 ± 0.21 ^aC^	7.75 ± 0.22 ^aC^
	300 ppm	15 min	6.57 ± 0.11 ^cA^	3.34 ± 0.11 ^cC^	4.30 ± 0.30 ^cB^	4.26 ± 0.35 ^cB^
		30 min	9.61 ± 0.29 ^bA^	7.52 ± 0.59 ^bB^	5.07 ± 0.44 ^bD^	6.01 ± 0.60 ^bC^
		45 min	11.55 ± 0.73 ^aA^	9.51 ± 0.76 ^aB^	6.98 ± 0.21 ^aC^	7.58 ± 0.64 ^aC^
	400 ppm	15 min	6.41 ± 1.11 ^cA^	4.24 ± 0.08 ^cB^	4.08 ± 0.50 ^cB^	4.52 ± 0.75 ^cB^
		30 min	10.66 ± 0.95 ^bA^	8.03 ± 0.84 ^bB^	5.82 ± 0.21 ^bC^	6.41 ± 0.48 ^bC^
		45 min	13.79 ± 0.55 ^aA^	9.85 ± 0.22 ^aB^	8.51 ± 0.81 ^aC^	8.48 ± 0.28 ^aC^
	500 ppm	15 min	5.87 ± 0.50 ^cAB^	4.82 ± 0.40 ^cBC^	4.13 ± 0.11 ^cC^	6.66 ± 1.63 ^bA^
		30 min	12.24 ± 0.33 ^bA^	9.36 ± 0.65 ^bB^	7.66 ± 0.62 ^bC^	9.07 ± 0.87 ^abB^
		45 min	14.46 ± 0.60 ^aA^	11.08 ± 0.33 ^aB^	9.79 ± 0.69 ^aB^	10.74 ± 1.37 ^aB^
Chicken breast	100 ppm	15 min	2.86 ± 0.21 ^cA^	1.05 ± 0.14 ^cB^	0.86 ± 0.16 ^cB^	2.89 ± 0.61 ^cA^
		30 min	3.92 ± 0.08 ^bB^	3.35 ± 0.49 ^bBC^	2.79 ± 0.68 ^bC^	5.21 ± 0.53 ^bA^
		45 min	5.46 ± 0.32 ^aB^	4.94 ± 0.21 ^aBC^	4.20 ± 0.47 ^aC^	7.35 ± 0.91 ^aA^
	200 ppm	15 min	2.31 ± 0.18 ^cB^	2.06 ± 0.43 ^cB^	1.77 ± 0.33 ^cB^	4.15 ± 0.67 ^cA^
		30 min	3.97 ± 0.28 ^bB^	5.52 ± 0.49 ^bA^	2.59 ± 0.17 ^bC^	6.29 ± 0.45 ^bA^
		45 min	6.05 ± 0.34 ^aC^	6.98 ± 0.59 ^aB^	4.88 ± 0.41 ^aD^	8.50 ± 0.26 ^aA^
	300 ppm	15 min	3.44 ± 0.13 ^cB^	2.86 ± 0.35 ^cB^	2.69 ± 0.53 ^cB^	5.84 ± 1.12 ^bA^
		30 min	7.10 ± 0.77 ^bA^	6.62 ± 0.39 ^bA^	4.43 ± 0.33 ^bB^	6.82 ± 0.64 ^bA^
		45 min	9.00 ± 0.63 ^aA^	7.78 ± 0.22 ^aB^	7.29 ± 0.02 ^aB^	9.13 ± 0.69 ^aA^
	400 ppm	15 min	4.84 ± 0.44 ^cA^	3.95 ± 0.63 ^cAB^	3.70 ± 0.20 ^cB^	3.80 ± 0.72 ^cAB^
		30 min	8.37 ± 0.30 ^bA^	6.45 ± 0.52 ^bB^	5.60 ± 0.58 ^bB^	8.03 ± 0.85 ^bA^
		45 min	11.48 ± 0.31 ^aA^	7.76 ± 0.39 ^aB^	6.77 ± 0.31 ^aB^	12.10 ± 1.59 ^aA^
	500 ppm	15 min	3.95 ± 0.31 ^cB^	4.01 ± 0.10 ^cB^	3.12 ± 0.56 ^cB^	5.97 ± 0.82 ^cA^
		30 min	8.73 ± 0.34 ^bB^	6.04 ± 0.23 ^bC^	6.47 ± 0.46 ^bC^	9.48 ± 0.23 ^bA^
		45 min	13.58 ± 0.23 ^aA^	8.86 ± 0.03 ^aB^	7.62 ± 0.20 ^aC^	13.20 ± 0.74 ^aA^
Chicken neck	100 ppm	15 min	3.57 ± 0.41 ^cAB^	3.77 ± 0.14 ^cA^	2.49 ± 0.20 ^cC^	2.84 ± 0.62 ^cBC^
		30 min	6.22 ± 0.39 ^bA^	5.79 ± 0.42 ^bA^	3.72 ± 0.11 ^bB^	5.65 ± 0.65 ^bA^
		45 min	8.08 ± 0.76 ^aA^	7.84 ± 0.17 ^aAB^	5.91 ± 0.34 ^aC^	7.09 ± 0.53 ^aB^
	200 ppm	15 min	4.60 ± 0.21 ^cA^	3.38 ± 0.11 ^cB^	2.34 ± 0.07 ^cC^	3.69 ± 0.79 ^cB^
		30 min	7.86 ± 0.45 ^bA^	4.70 ± 0.19 ^bC^	4.43 ± 0.41 ^bC^	6.12 ± 0.48 ^bB^
		45 min	10.51 ± 0.23 ^aA^	8.24 ± 0.68 ^aB^	6.31 ± 0.62 ^aC^	9.01 ± 0.68 ^aB^
	300 ppm	15 min	7.01 ± 0.58 ^cA^	3.92 ± 0.75 ^cB^	3.58 ± 0.70 ^bB^	4.32 ± 0.18 ^cB^
		30 min	10.07 ± 0.52 ^bA^	6.54 ± 0.13 ^bB^	6.97 ± 0.65 ^aB^	7.45 ± 0.41 ^bB^
		45 min	12.95 ± 0.18 ^aA^	8.76 ± 0.53 ^aB^	7.85 ± 0.59 ^aC^	9.22 ± 0.38 ^aB^
	400 ppm	15 min	7.30 ± 0.54 ^cA^	4.38 ± 0.22 ^cC^	3.72 ± 0.47 ^cC^	6.49 ± 0.41 ^bB^
		30 min	11.11 ± 1.20 ^bA^	7.57 ± 0.27 ^bC^	6.06 ± 0.06 ^bD^	9.26 ± 0.49 ^aB^
		45 min	13.26 ± 1.17 ^aA^	10.75 ± 0.29 ^aB^	8.73 ± 0.35 ^aC^	10.21 ± 0.85 ^aB^
	500 ppm	15 min	8.47 ± 1.01 ^bA^	5.50 ± 0.81 ^cB^	5.50 ± 1.07 ^bB^	6.42 ± 0.12 ^cB^
		30 min	10.48 ± 1.14 ^bA^	8.75 ± 0.49 ^bAB^	7.22 ± 1.70 ^abB^	9.68 ± 0.23 ^bA^
		45 min	13.28 ± 1.39 ^aA^	11.41 ± 0.56 ^aA^	8.84 ± 1.76 ^aB^	11.56 ± 0.15 ^aA^

Values are presented as the mean ± standard deviation (*n* = 3). ∆E* = ((∆L*)^2^ + (∆a*)^2^ + (∆b*)^2^)^0.5^. Different uppercase letters in the same row (A–D) indicate significant differences (*p* < 0.05) according to Duncan’s multiple range test. Different lowercase letters in the same column (a–c) indicate significant differences (*p* < 0.05) according to Duncan’s multiple range test.

## Data Availability

The original contributions presented in the study are included in the article, further inquiries can be directed to the corresponding author.
